# Molecular Biosensors for Electrochemical Detection of Infectious Pathogens in Liquid Biopsies: Current Trends and Challenges

**DOI:** 10.3390/s17112533

**Published:** 2017-11-03

**Authors:** Susana Campuzano, Paloma Yáñez-Sedeño, José Manuel Pingarrón

**Affiliations:** Departamento de Química Analítica, Facultad de Ciencias Químicas, Universidad Complutense de Madrid, E-28040 Madrid, Spain; yseo@quim.ucm.es

**Keywords:** electrochemical affinity biosensors, bacteria, viruses, immunosensors, nucleic acid-based sensors, liquid biopsies

## Abstract

Rapid and reliable diagnosis of infectious diseases caused by pathogens, and timely initiation of appropriate treatment are critical determinants to promote optimal clinical outcomes and general public health. Conventional in vitro diagnostics for infectious diseases are time-consuming and require centralized laboratories, experienced personnel and bulky equipment. Recent advances in electrochemical affinity biosensors have demonstrated to surpass conventional standards in regards to time, simplicity, accuracy and cost in this field. The tremendous potential offered by electrochemical affinity biosensors to detect on-site infectious pathogens at clinically relevant levels in scarcely treated body fluids is clearly stated in this review. The development and application of selected examples using different specific receptors, assay formats and electrochemical approaches focusing on the determination of specific circulating biomarkers of different molecular (genetic, regulatory and functional) levels associated with bacterial and viral pathogens are critically discussed. Existing challenges still to be addressed and future directions in this rapidly advancing and highly interesting field are also briefly pointed out.

## 1. Introduction

Pathogen infections are responsible for thousands of deaths and an enormous burden of morbidity worldwide. Therefore, they are considered as major concern for global health. Despite the availability of antibiotics and antiviral therapies, bacterial and viral infections are often misdiagnosed or diagnosed with an unacceptable delay [1]. Therefore, the accurate and early identification of patients requiring treatment using simple, fast and affordable diagnostic tests is a very important issue for effective treatment and control. In addition, the possibility of a reliable diagnosis of these infections in a minimally invasive way should allow targeting of anti-infective therapy more effectively, while significantly reducing the number of unnecessary procedure-associated complications.

The diagnosis of a patient is usually performed by an authorized person in a quick manner, paying attention to the symptoms of the patient and particular prevalence of infections at the time and location. In addition, since the concentration of the infectious pathogen is not accurately known, an antibiotic is sometimes unnecessarily administered or done at inappropriate doses. Therefore, in order to allow the application of therapy in an early and efficient way using doses correlated with the actual status of the infection, the development of simple and inexpensive methodologies, able to be easily translated to point-of-care testing (POCT), are highly demanded to determine the target infectious pathogen in an accurate and selective way [2,3,4,5]. 

Conventional tests employing cell culture, and serological tests last from two days up to two weeks, and therefore are not suited for fast detection. Moreover, some viruses are hard or impossible to cultivate [5]. On the other hand, molecular tests, such as reverse transcription-polymerase chain reaction (RT-PCR), and enzyme-linked immunosorbent assay (ELISA) are sensitive and rapid, but they are expensive, complicated to implement and require specialized equipment and trained users. Accordingly, with the aim of reducing the social and economic costs, the development of rapid diagnostic tests with high throughput and the possibility of multiple target detection, accuracy (in terms of specificity and sensitivity), ease of use, affordability and suitability for on-site use in the field is an important research subject. Such requirements are met by portable stand-alone electrochemical molecular biosensors. A molecular biosensor is a device that uses specific biochemical reactions mediated by isolated enzymes, immunosystems, tissues, organelles or whole cells to detect chemical compounds usually by electrical, thermal or optical signals [6]. Electrochemical molecular biosensors are able to facilitate rapid detection and diagnosis at the POC, which make them particularly useful for an early and unequivocal diagnosis, and to prevent further disease spread [1,7].

## 2. Infectious Pathogens: Bacteria and Viruses 

Pathogens are microorganisms that cause infectious disease. Bacteria and viruses are the main pathogens. Bacteria release toxins, and viruses damage cells. White blood cells destroy pathogens by ingestion or producing antibodies against them and antitoxins to neutralise toxins. In vaccination, pathogens are introduced into the body in a weakened form which causes the body to produce enough white blood cells to protect itself against the pathogens and not get diseased. Antibiotics are effective against non-resistant bacteria, but not against viruses. 

The efficient control of the spread of disease and the improvement of patient outcomes are directly related to the early, rapid and reliable detection of infectious pathogens. In fact, it has been reported that patient survival diminishes about 8% every hour a blood infection is not properly treated [4,8]. Moreover, the fast identification of the cause of an infection plays a determinant role in controlling antibiotic resistance rates and optimizing antibiotic administration. Since the analyses of bacteria and viruses are performed in different body fluids, and the concentration levels of the infectious pathogens depend remarkably on the type of biofluid to be analyzed, developed methods should consider these differences to implement reliable determination [4].

The diagnosis and evaluation of viral infections are mostly based on the detection of specific nucleic acid sequences of the target virus. In this context, it is important to note that levels of blood-borne viruses are typically ~10^6^ infectious units (IUs) per mL of blood, which corresponds to a femtomolar concentration of viral RNA [4]. For instance, Dengue and Zika are viruses transmitted by mosquitos that cause tropical diseases [9]. The levels at which dengue viral RNA can be detected in biological samples (blood, saliva or urine) are between 10^3^ and 10^6^ copies per milliliter [10,11,12]. In the case of Zika, the corresponding levels range from 10^2^–10^5^ copies per milliliter in plasma or urine [13]. However, these are one thousand times higher in semen, which gives an idea of the capacity for sexual transmission of this infection [14]. *Flavivirus influenza* loads are typically 10^6^–10^9^ in aspirate samples and as low as 10^3^ viral particles mL^−1^ in throat and nasal swabs [4,15,16,17].

Regarding bacterial infections, the unequivocal identification of a wide variety of Gram-positive and Gram-negative bacteria including *Escherichia coli*, *Staphylococcus aureus* and *Klebsiella*, along with drug-resistant strains in the bloodstream of an infected patient are challenging applications, since the presence of as few as 1–10 colony-forming units (cfus) of bacteria in a milliliter of blood are enough to cause a life-threatening infection [18]. If an rRNA is used as a target, it should be present at a copy number up to 10^3^ or 10^4^ which corresponds to detect a subfemtomolar concentration of RNA in the sample with an overwhelming excess of non-target RNA. This constitutes a big challenge for implementing direct detection methods and the main reason why molecular-level analysis is typically performed after bacterial culture enrichment [4].

Regarding tuberculosis (TB), it is important to highlight the increase in this type of drug-resistant infection in places where access to clinical trials is difficult. In this case, the usual sample is sputum, in which the levels are elevated (10^7^–10^8^ cells per milliliter) [19,20], although it is a viscous sample whose analysis offers a high difficulty [5].

*Escherichia coli*, *Staphylococcus saprophyticus*, *Klebsiella pneumoniae*, *Proteus mirabilis* and *Pseudomonas aeruginosa* are the main pathogens causing urinary tract infections (UTIs). Methods for rapid and sensitive detection of these bacteria are required because of the increasing emergence of resistance to drugs from these common infections, which extend to second and third line antibiotics [21]. UTIs are usually detected in urine which is considered positive if it contains more than 10^5^ cfu mL^−1^ [22]. Importantly, the low concentrations of other potential interfering substances present in this sample, compared with other types of biological samples [4], means the specificity of methods for UTIs detection are not cruicial. 

## 3. Conventional Methods for the Determination of Infectious Pathogens

In order to diagnose common infectious diseases, various steps should be performed. Firstly, biological samples should be collected and transported from the point where they are taken to the laboratory. Then, samples are processed, analyzed by applying the appropriate protocols, and the results reported. These steps, which must be performed by qualified personnel, usually take several days, and the results are communicated to the patient by the clinician. The delay in notifying the results to make further decisions is one of the disadvantages of this procedure. It has been demonstrated that these shortcomings become more evident in places with few health resources and have led, for example, to the reckless use of antimicrobials [4]. 

Gold-standard techniques for bacterial and viral infections diagnostics include microscopy, plating and culturing methods, nucleic acid-based techniques and immunological assays. Among them, immunological or molecular-based assays including DNA hybridization and polymerase chain reaction (PCR) methods should be highlighted. Immunological assays rely on the specificity of the antigen–antibody recognition and are suitable for the detection of a whole range of agents affecting global health. In particular, ELISAs, which are approved by regulatory agencies and commercially available, are the most common methods used for virus-specific antigens determination. However, they are time-consuming and multistage, provide relatively low sensitivity, may produce false negative results and their performance depends strongly on operator skills [4,5,7,23,24]. 

Nucleic acid-based detection may be more specific and sensitive than immunological methods. It is based on the detection, by performing efficient hybridization with complementary oligonucleotides, of DNA or RNA sequences characteristic of the target pathogen which allow unequivocal identification. In general, these specific DNA and RNA sequences are the preferred targets for infectious disease testing, as they directly correspond to the presence of an active infection [4]. Hybridization assays prove to be especially powerful in the rapid and sensitive detection of synthetic short oligonucleotides. However, when faced with genomic DNA or total RNA, the length and complexity of the target, together with the sample media, limit their application. Even in the cases where hybridization assays show high enough analytical sensitivity to detect very low amounts of DNA and RNA, sample pretreatment to restrict the size of the target is usually required to allow an efficient hybridization. A useful option is to couple the hybridization assays with amplification methods, which contribute to both specifically amplify the target and restrict its size [25]. Indeed, this high sensitivity made nucleic acid amplification widely used for the detection of pathogen infections. Despite these advantages, these detection methods are still relatively complex, technically demanding and costly, and require centralized laboratories, trained personnel and bulky specialized equipment with regular maintenance or reliable electric supply, and therefore costly and not available in all countries and settings [1,5].

In this sense, electrochemical biosensors coupled to inexpensive field-portable and programmable battery-operated instrumentation offer exciting alternatives as rapid and cost-effective analytical strategies easy to be handled by unskilled personnel. 

This review, through selected examples, aims to demonstrate that electrochemical affinity biosensors (mainly ADN- and immunosensors) offer considerable promise for obtaining reliable information about infectious diseases caused by bacterial and viral pathogens in a fast, simple, cheap and minimally invasive manner. Only these methods focus on the determination of circulating biomarkers and have demonstrated applicability in the analysis of biofluids (mainly blood, serum, saliva, urine, sputum and pleural fluid). They are discussed and classified regarding the type of pathogen determined, bacteria or viruses. Main bottlenecks and future research directions involved are also pointed out at the end of the article.

## 4. Electrochemical Affinity Biosensing of Infectious Pathogens in Liquid Biopsies

Electrochemical biosensors have emerged as reliable molecular sensing devices suitable for the detection of pathogens in liquid biopsies. Short assay time, simple handling, low cost, small sample requirement, possibility of multiplexing and miniaturization and good performance in complex samples with minimal pre-treatments justify their increasing growing and suitability for POC applications [7,21]. 

The incorporation of a wide variety of nanomaterials with large surface area, abundant binding sites and unique catalytic activity, conductivity and biocompatibility, have led to the development of electrochemical affinity biosensors which meet the requirements for infectious pathogen determination in biofluids at the required levels [26,27]. These nanomaterial-based electroanalytical bioplatforms have demonstrated enhanced sensitivity, selectivity, minimal interferences from biological matrices and prolonged stability. Nanomaterials such as CNTs and AuNPs have been used as electrode modifiers and advanced labels. Moreover, electrodes modified with tetrahedral surface DNA nanostructures have been reported in an electrochemical immunosensing strategy for *S. pneumoniae* determination [28]. 

The main bioreceptors used in the construction of electrochemical affinity biosensors for infectious pathogens include specific single-stranded (ss) oligonucleotide, peptide sequences and antibodies. 

The required sensitivity is achieved, in many cases, by coupling of nucleic acid-based biosensors with different nucleic acid amplification strategies, ranging from the conventional PCR, of limited use in centralized laboratories, to more recent and attractive alternatives involving isothermal amplification strategies. Isothermal amplification strategies involve either the use of strand displacement polymerases combined with especially designed primers or probes, as is the case in loop-mediated amplification (LAMP) and rolling-circle amplification (RCA), or mimicking in vivo DNA replication utilizing helicases for DNA unwinding (HDA). Indeed, the false positives (arising from primer-dependent artifacts and non-specific amplification) of these simplest isothermal amplification strategies are overcome by their coupling with electrochemical nucleic acid sensors due to the additional selectivity provided by the fully complementary specific probe oligonucleotide immobilized onto the electrode surface. Hybridization chain reaction (HCR), an isothermal and enzyme-free amplification strategy, has been used along electrochemical DNA sensors for the detection of bacterial infections. The use of isothermal amplification strategies reduces the cost and amplification time without sacrificing sensitivity, and can be carried out in clinical specimens because they exhibit good tolerance to the most common PCR inhibitors [29]. Interestingly, without the need for complex control systems of temperature [30], their integration into portable and cheaper devices to perform analysis in low-resource settings or at the point-of-need is greatly simplified. At this point, it is worth to mention that very recently the Lobo-Castañón’s research group has reported, for the first time, the use of on-surface helicase-dependent amplification at ITO surfaces to quantify electrochemically very small amounts of genomic DNA extracted from *Salmonella* [31]. Attractive electrochemical nucleic acid sensors have been reported so far for non-invasive bacterial infections detection coupled with PCR [32,33,34], HDA [25,31,34], RPA [35] and HCR [36]. 

Electrochemical biosensors described to date for bacterial infections [28,32,33,34,35,37,38,39,40,41,42,43,44,45] comprise a rather limited number of immunosensors using antibodies able to detect the whole bacteria (*Streptococcus pseudopneumoniae*, *Escherichia coli* and *Vibrio cholerae*) [36,37,38] or specific surface bacterial proteins (PspA peptide) [28], and a wide variety of nucleic acid sensors targeting specific bacterial genes used along with different nucleic acid and signal amplification strategies [32,33,34,35,39,40,41,42,43,44,45]. 

Regarding viral infection determination [46,47,48,49,50,51,52,53,54,55,56,57,58,59], electrochemical biosensing approaches have been focused mainly on the detection of:

(i) the whole virus such as human enterovirus 71 [55];
(ii) specific viral proteins (p24 from HIV [46,47,48,49] and NS1 from Dengue [54]);
(iii) autoantibodies against specific viral antigens (pseudorabies virus [50], HIV-1 and HIV-2 [51,56] and hemagglutinin, HA, from avian influenza virus H5N1 [52,53]) and
(iv) characteristic DNA sequences (Dengue virus serotype 3, DENV-3, [57] and high-risk human papillomavirus, hrHPV, strains HPV-16 and HPV-18 [58]).

The reported methods imply the development of immunosensors for the detection of specific viral proteins and intact virus, DNA sensors for targeting characteristic DNA sequences and biosensor involving the immobilization of the specific viral antigens or peptides for the determination of the humoral immune response against viral infections. 

The following sections show that electrochemical affinity biosensors are emerging technologies suitable for the detection of bacterial and viral infections in a non-invasive way through the determination of the whole pathogen, pathogen specific biomolecules, such as surface proteins/antigens or antibodies against them, nucleic acids or virulence factors.

### 4.1. Minimally Invasive Electrochemical Biosensing of Bacterial Pathogens

A disposable amperometric magnetoimmunosensor, using Protein A-modified magnetic microbeads and gold screen-printed electrodes (Au/SPE), was developed for the selective determination of *Streptococcus pneumoniae* [37]. A sandwich configuration using the same antibody for capture and detection (in this case conjugated with HRP) was employed. MBs bearing the sandwich immunocomplexes were magnetically captured on the surface of tetrathiafulvalene (TTF)-modified Au/SPE and the amperometric signal measured at −0.15 V vs. the silver pseudoreference electrode of the Au/SPE in the presence of H_2_O_2_ was used as transduction signal. A LOD of 3.0 × 10^3^ cfus without pre-enrichment steps was accomplished in 3.5 h from sampling to measurement. The LOD was calculated according to the 3 × s_b_/m criterion where s_b_ was the standard deviation of the blank and m was the analytical sensitivity. The usefulness of the method was proved by analyzing undiluted human urine samples inoculated with the target bacteria. 

**Figure 1:**
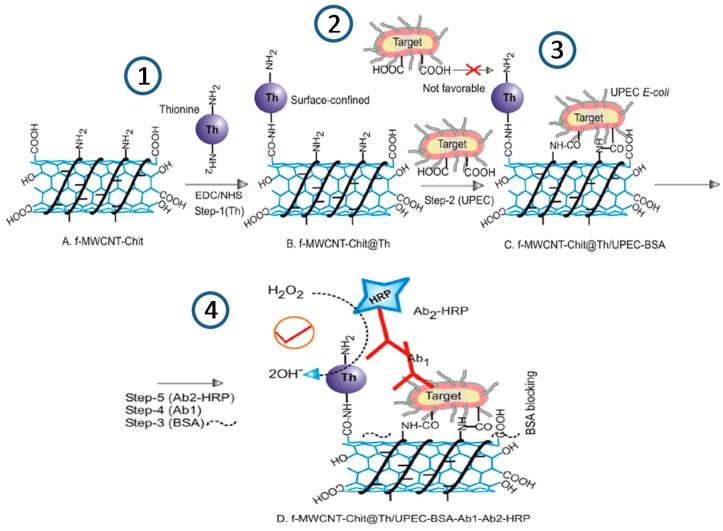
Schematic illustration of the preparation of an electrochemical immunosensor for the determination of *Escherichia coli* using a f-MWCNT-Chit@Thionine-chemically modified electrode (step 1); by immobilization of cells (step 2), BSA (step 3), Ab1 and Ab2 HRP (step 4). Reprinted and adapted from [38] with permission.

Gayathri et al. [38] developed an electrochemical immunosensor for the detection of uropathogenic *Escherichia coli* (UPEC) using as scaffold a glassy carbon electrode (GCE) modified with a thionine dye immobilized chitosan/functionalized-MWCNT composite (GCE/f-MWCNT-Chit@Th) (Figure 1). The bacteria were covalently immobilized on this surface and labeled with a primary antibody and an HRP-tagged secondary antibody. By monitoring the H_2_O_2_ reduction reaction using cyclic voltammetry, the immunosensor showed excellent linearity between 10^2^ and 10^9^ cfus mL^−1^, which is a range of practical interest to detect urinary bacterial infections. The immunosensor also exhibited a successful specificity towards other bacteria and applicability to the analysis of spiked raw urine samples.

**Figure 2:**
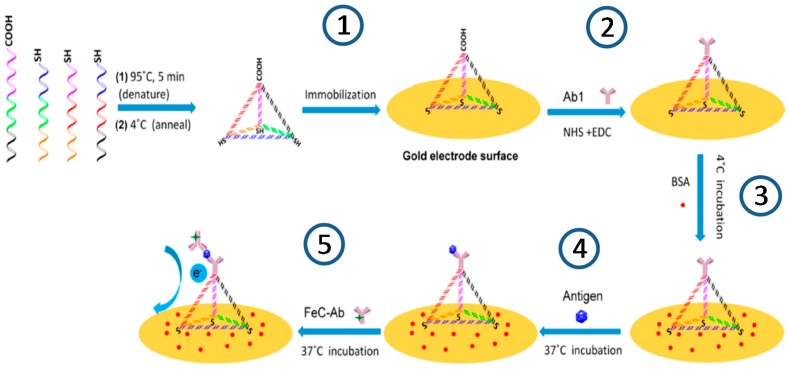
Schematic illustration of the construction of the DNA tetrahedron (DNATH)-based electrochemical immunosensor using a ferrocene-conjugated detector antibody for PspA peptide determination: (1) assembling of DNATH onto the electrode surface; (2) covalent immobilization of antibody with upright orientation; (3) blocking step with BSA; (4) incubation with antigen, and (5) labeling of the antigen with FeC-antibody and electrochemical detection. Reprinted from [28] with permission.

A sandwich electrochemical immunosensor for rapid detection of pneumococcal surface protein A (PspA) peptide was proposed by immobilizing the capture antibody onto a gold electrode nanostructured with a DNA tetrahedron (DNATH) and using the same antibody conjugated with ferrocene (FeC-Ab) as a detector (see Figure 2) [28]. The concentration of the antigen is followed by measuring the increase in the square wave voltammetric (SWV) signal corresponding to the oxidation of Fc in the FeC-AbC. This DNA nanostructured-immunosensor demonstrated feasibility to perform the determination using both purified PspA peptide and *Streptococcus pneumoniae* lysates with linear ranges of 0–8 ng mL^−1^ and 5–100 cfus mL^−1^, and LODs (S/N = 3) of 0.218 ng mL^−1^ and 0.093 cfu mL^−1^, respectively. The immunosensor also demonstrated applicability to the determination in uncultured samples obtained from the upper respiratory tract, mouth and axilla of the same individual. 

**Figure 3:**
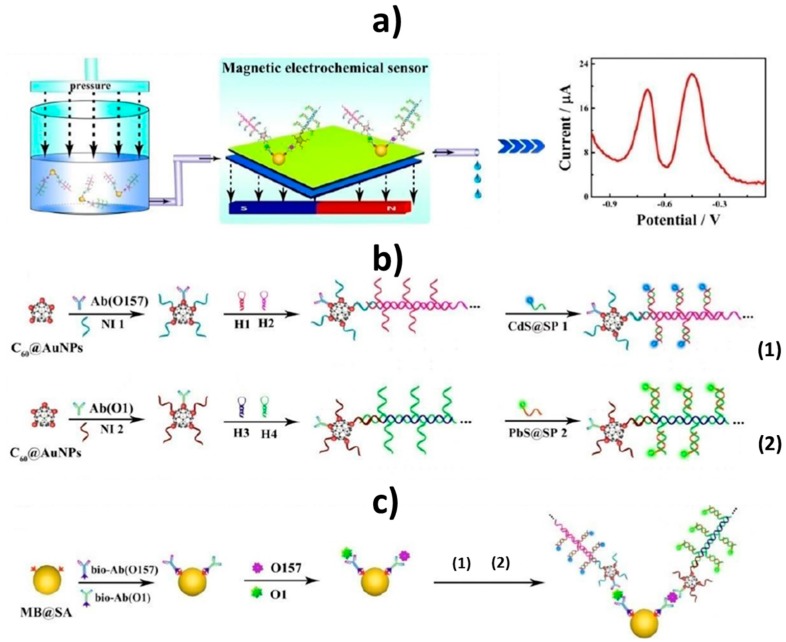
Schematic display of the electrochemical biosensor developed for the simultaneous detection of *Escherichia coli* O157:H7 and *Vibrio cholerae* O1. Schematic diagram of the simultaneous electrochemical system (**a**); preparation of the bioconjugate- and nanoparticle-labeled antibodies (**b**); schematic diagram of the immunoassay on magnetic beads (**c**). Abbreviations: Ab(O157) or Ab(O1), antibodies for *Escherichia coli* O157:H7 or *Vibrio cholerae* O1; Bio-Ab, biotinylated antibodies; H1–4, hairpin nucleotide for HCR reaction; MB@SA, streptavidin-coated magnetic beads; NI1–2, nucleotide initiator; SP1–2, signal probe. Reprinted and adapted from [36] with permission.

Li et al. [36] developed an electrochemical strategy using multifunctional nanoconjugates for sensitive simultaneous detection of *Escherichia coli* O157:H7 and *Vibrio cholerae* O1. The method utilized the specific immune recognition of different pathogenic bacteria by multifunctional nanoconjugates and subsequent signal amplification. The biotinylated specific capture antibodies for *Escherichia coli* O157:H7 and *Vibrio cholerae* O1 were attached to streptavidin-coated magnetic beads. The detection antibodies were labeled with exponentially large amounts of signal indicators (CdS and PbS nanoparticles)-labeled signal probes via C_60_@AuNPs as nanocarriers and HCR amplification (Figure 3). Enhanced differential pulse voltammetric responses were proportional to the concentration of *Escherichia coli* O157:H7 and *Vibrio cholerae* O1 from 5 × 10^1^ to 1 × 10^6^ cfus mL^−1^, and allowed achieving LODs (3 × s_b_/m) of 39 and 32 cfus mL^−1^, respectively. The feasibility of the method to measure the concentration of both pathogenic bacteria in a single run was demonstrated by analysis in human stool and water samples providing results in agreement with the plate count method.

Wu et al. [39] developed a very sensitive electrochemical DNA sensor for the specific determination of *Escherichia coli* 16S ribosomal RNA (rRNA). The biosensor consisted of a 16-Au electrode array prepared by photolithography, and modified with a ternary self-assembled monolayer of a thiolated specific DNA capture probe, mercaptohexanol (MCH) and dithiothreitol (DTT). Using a sandwich hybridization format with a fluorescein (FITC)-modified detector probe labeled in a final step with antiFITC-HRP Fab fragments and chronoamperometric detection using the H_2_O_2_/3,3′,5,5′-tetramethylbenzidine (TMB) system, the method allowed remarkably low LODs down to 40 zmol of synthetic target and only 1 cfu *Escherichia coli* per sensor in 45 min. The usefulness of this bioplatform was demonstrated by analyzing 18 real uropathogenic clinical isolates, showing 100% sensitivity and specificity. 

**Figure 4:**
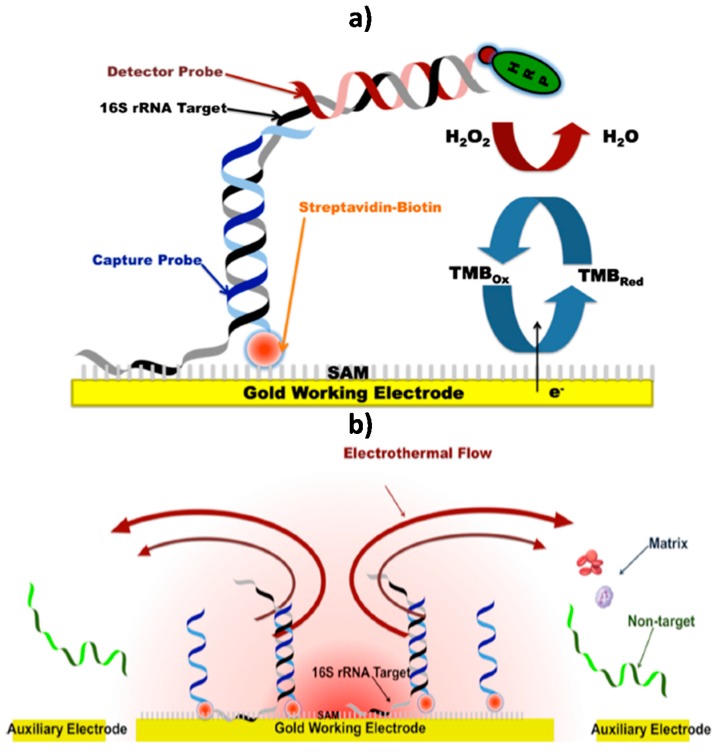
Schematic display the electrochemical DNA platform developed (**a**) and the AC electrokinetics-enhanced hybridization process (**b**) for bacterial 16S rRNA identification for rapid AST required in diagnosis of acute bacterial infections. Reprinted from [40] with permission.

A rapid antimicrobial susceptibility test (AST) that combines phenotypic culture of bacterial pathogens in physiological samples and electrochemical genotypic molecular detection was developed by Liu et al. [40] by targeting bacterial 16S rRNA, and using a very similar approach and the same chronoamperometric transduction in the presence of TMB/H_2_O_2_, but immobilizing a biotinylated-specific capture probe onto the same Au electrode array pre-coated with an alkanethiol SAM and streptavidin (Figure 4a). The authors demonstrated the use of alternating current (AC) electrokinetic fluid motion and Joule heating-induced temperature elevation to enhance the sensor signal and minimize the matrix effect. Under the optimized electrokinetic conditions, direct detection of bacterial pathogens in blood culture without prior purification, sample preparation and nucleic acid extraction steps was claimed (Figure 4b). The applicability of the electrokinetic-enhanced biosensing platform was checked through the rapid determination of the antibiotic resistance profiles of *Escherichia coli* clinical isolates in blood cultures. The same authors developed a multiplexed electrochemical biosensor for bloodstream infection diagnosis by detecting the species-specific sequences of the 16S rRNA of bacteria including *Staphylococcus aureus*, *Escherichia coli*, *Pseudomonas aeruginosa* and *Proteus mirabilis* in physiological samples without pre-amplification [41]. In this case, the DNA sensing platform was prepared using a ternary layer composed of thiolated capture probe, hexanedithiol (HDT) and MCH. The feasibility of this multiplexed biosensor for diagnosis of a bloodstream infection was demonstrated by identifying bacterial clinical isolates in spiked whole blood samples, providing results with 100% agreement with microbiological analysis.

A rapid urine test for detection of urogenital *Schistosomiasis* was developed by Mach et al. [42] using a similar approach. In this case the *Schistosoma haematobium* 16S rRNA was sandwiched between a specific thiolated DNA capture probe self-assembled onto a gold electrode and a FITC-labeled detector probe. The hybridization reaction was monitored upon incubation with a HRP-conjugated anti-FITC antibody by chronoamperometry using TMB/H_2_O_2_ . The DNA sensor allowed the detection of 0.53 ng mL^−1^ total RNA isolated from *Schistosoma haematobium* egg and ~30 *Schistosoma haematobium* eggs per mL of human urine.

**Figure 5:**
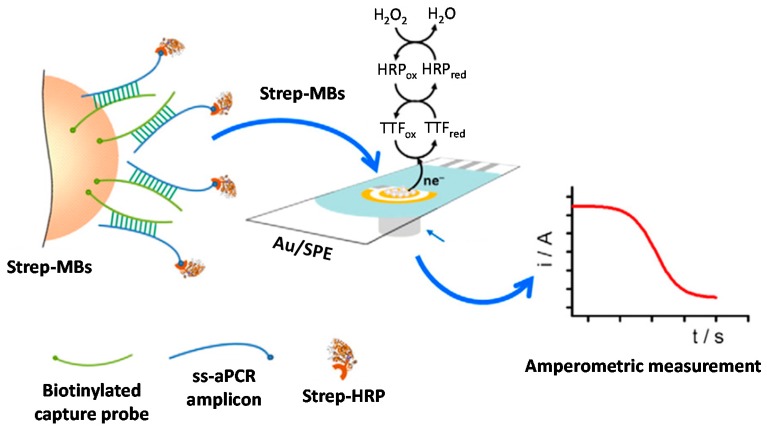
Schematic display of the amperometric DNA sensor developed for *Streptococcus pneumoniae* determination in connection with MBs and aPCR by targeting a specific fragment of *lytA* gene coding sequence. Reprinted from [33] with permission.

A disposable amperometric DNA sensor involving the use of MBs and asymmetric polymerase chain reaction (aPCR) was developed for the detection of *Streptococcus pneumoniae* by targeting a specific region of the pneumococcal *lytA* gene [32]. In this approach, commercial Strep-MBs modified with a specific biotinylated DNA probe were used as microcarriers to selective capture the predominantly 235-base ss-amplicon generated by direct aPCR (daPCR) from bacterial cultures. The biotinylated duplexes captured at the Strep-MBs were labeled with a Strep-HRP polymer to perform amperometric detection upon adding H_2_O_2_ at TTF-Au/SPEs (Figure 5). The method allowed obtaining LODs (3 × s_b_/m) of 5.1 nM (20-mer synthetic target DNA) and 1.1 nM (ss-aPCR amplicon), as well as obtaining daPCR amplicons with as few as 2 cfus of *Streptococcus pneumoniae*. The methodology permitted discrimination between *Streptococcus pneumoniae* and closely related streptococci, such as *Streptococcus mitis,* and between blood and urine samples non-inoculated and inoculated at 10^3^ cfus mL^−1^ of the target bacteria. Three years later, the same authors demonstrated the successful validation of the developed DNA sensor with 109 clinical samples obtained from skin, abscesses, sputum, purulence, blood, swabs taken from throat, ear or conjunctiva, nasal, tracheal or bronchial aspirates, pleural fluid and bronchoalveolar washes [33].

Yamanaka et al. [43] designed an electrochemical strategy to determine *Porphyromonas gingivalis* (a bacterium causing periodontal disease) by mixing bisbenzimidazole trihydrochloride used as electrochemical intercalator with the amplicons obtained by direct PCR. The peak current of the indicator, measured by linear sweep voltammetry, decreased in the presence of amplicons over the 10^0^–10^4^ cell range due to slower diffusion of the indicator after intercalation into the amplified DNA. The analysis of saliva samples demonstrated that the determination of the obtained dPCR reflected clearly the periodontal disease degree.

**Figure 6:**
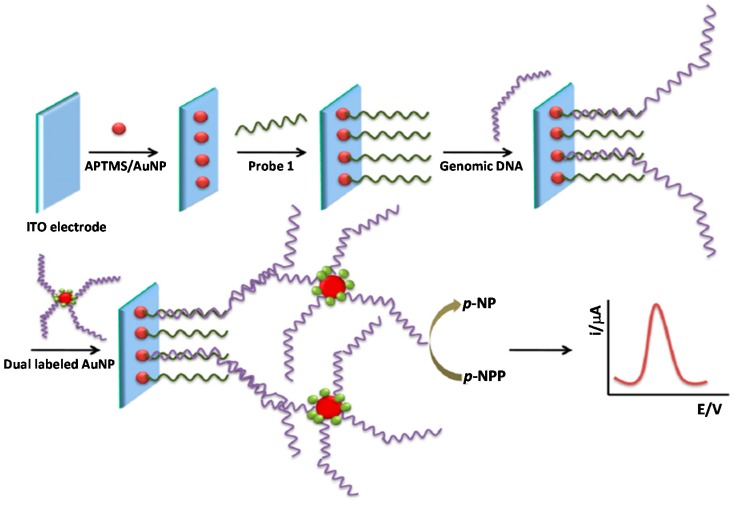
Schematic display of the electrochemical DNA biosensor developed for *Mycobacterium* sp. determination using an indium tin oxide (ITO) electrode modified with a specific capture probe and AuNPs dually labeled with detector probe and AP. Reprinted from [44] with permission.

Thiruppathiraja et al. [44] developed a DNA biosensor for *Mycobacterium* sp. detection by sandwiching the target genomic DNA between a specific probe immobilized on a ITO electrode modified with a (3-aminopropyl)trimethoxysilane SAM and detector probe/alkaline phosphatase (AP)-dually labeled AuNPs for amplification purposes (Figure 6). Through DPV determination of the para-nitrophenol generated by AP hydrolysis of para-nitrophenol phosphate, this method allowed a LOD of 1.25 ng mL^−1^ genomic DNA to be achieved and was successfully applied to the determination of clinical sputum samples.

**Figure 7:**
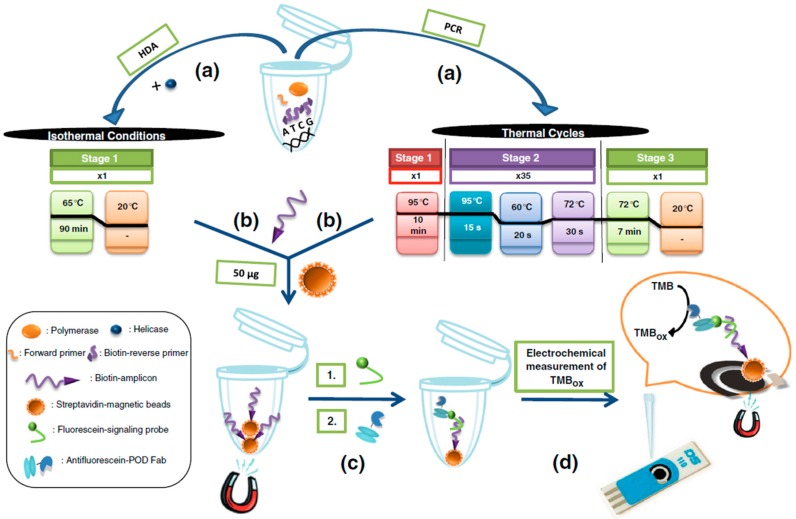
Schematic illustration of the steps involved in the determination of *Mycobacterium tuberculosis* using an electrochemical MBs-based DNA assay coupled to aHDA and aPCR amplifications (**a**). After immobilization of the biotinylated ss-amplicon resulting from the amplification onto the surface of Strep-MBs (**b**) and further hybridization and labeling with the FITC-detector probe and HRP-antiFITC antibody (**c**), the electrochemical detection was performed (**d**). Reprinted from [34] with permission.

Lobo-Castañón´s group also described a very interesting electrochemical DNA sensing strategy for *Mycobacterium tuberculosis* determination by combining the use of MBs and chronoamperometric detection at SPCEs with asymmetric helicase-dependent DNA amplification (aHDA) [25]. The method involved the immobilization of the biotinylated ss-amplicon (an 84-base-long ss-DNA fragment of the insertion sequence IS6110 characteristic of *Mycobacterium tuberculosis*) onto the surface of Strep-MBs, its hybridization with an FITC-detector probe and further labeling with an HRP-conjugated antiFITC antibody (Figure 7). The electrochemical transduction was performed by chronoamperometry using the H_2_O_2_/TMB system upon magnetically capturing the modified MBs onto the SPCE surface. The method allowed obtaining a wide linear range (1 aM-1 fM) and a LOD of 0.5 aM for the synthetic target DNA in less than 4 h. One year later, the same group [34] compared the performance of this method with that achieved by using aPCR (see Figure 7). They demonstrated the same dynamic range (between 30 and 3000 copies) and similar LODs (3 × s_b_/m) of 0.4 aM (aPCR) and 0.5 aM (aHDA) with both amplification approaches. These results demonstrated the HDA method was a viable and simpler alternative to PCR, since the fact of not requiring a thermocycler permits it to be adapted easily to a broad range of settings. This electrochemical DNA sensor coupled with both amplification strategies showed successful results in the detection of *Mycobacterium tuberculosis* in sputum, urine and pleural fluid samples. 

**Figure 8:**
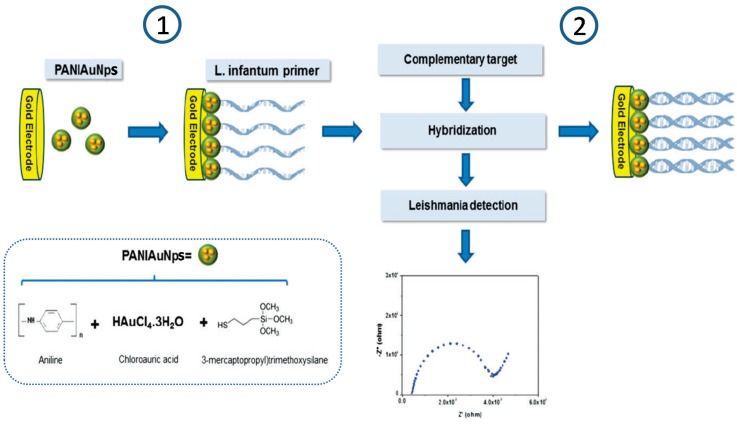
Schematic display of the DNA impedimetric sensor developed for *Leishmania infantum* determination using a gold electrode modified with PANIAuNPs: (1) modification of a gold electrode with PANIAuNPs and immobilization of *L. infantum* primer and (2) hybridization with complementary target and impedimetric detection. Inset: reagents used for the preparation of PANIAuNPs. Reprinted from [45] with permission.

García et al. developed an impedimetric DNA sensor for *Leishmania infantum* selective determination [45] by immobilizing a specific DNA probe onto a gold electrode previously coated with a layer of 3-mercaptopropyltrimethoxysilane on a polyaniline matrix containing AuNPs (PANIAuNPs) (Figure 8). By monitoring the hybridization using EIS in the presence of Fe(CN)_6_^3−/4−^, this sensor was able to determine different concentrations of *Leishmania infantum* genomic DNA (1–4 ng mL^−1^) and to analyze contaminated canine serum samples. 

**Figure 9:**
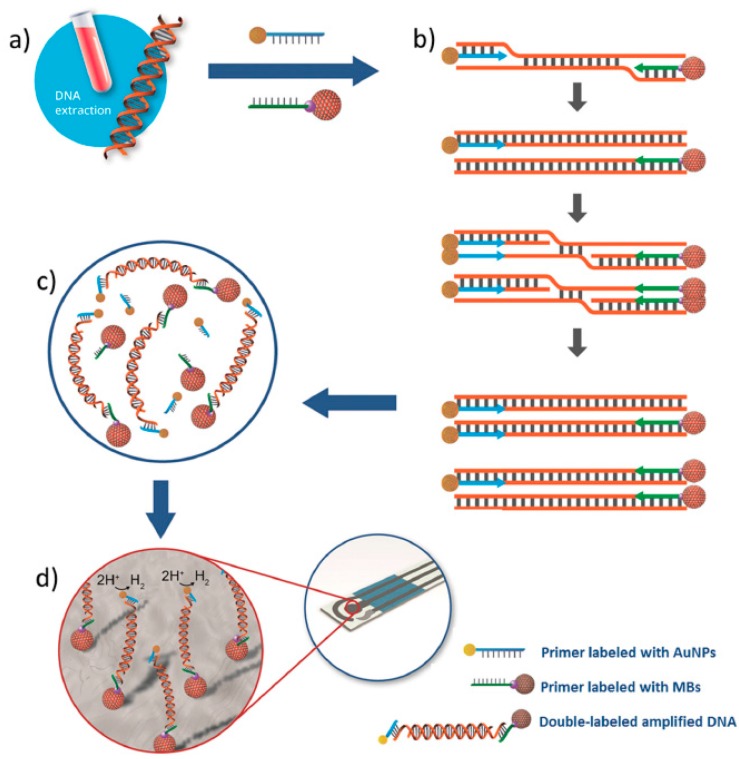
Scheme of the electrochemical method developed to determine *Leishmania* DNA by combining RPA and primers labeled with AuNPs and MBs comprising the following steps: DNA extraction from the blood sample (**a**), isothermal RPA using AuNPs and MBs labeled primers (**b**), magnetic capture of the MB and AuNPs dually labeled amplicons on the SPCE (**c**) and chronoamperometric detection of the hydrogen evolution reaction by the AuNPs (**d**). Reprinted from [35] with permission.

Another interesting methodology for the determination of *Leishmania* DNA was proposed by combining the use of isothermal recombinase polymerase amplification (RPA), primers labeled with MBs and AuNPs and chronoamperometric detection at SPCEs (Figure 9) [35]. The double-labeled amplicons were magnetically captured onto the SPCE, and the electrocatalytic activity of the AuNPs towards the hydrogen evolution reaction measured by chronoamperometry. The method exhibited a linear relationship between the current measured and the logarithm of parasite concentration in the range of 0.5–500 parasites mL^−1^ of blood, a LOD (S = B + 3σ) of 0.8 parasites mL^−1^ of blood and clear discrimination between healthy and infected dogs’ blood samples.

### 4.2. Minimally Invasive Electrochemical Biosensing of Viral Pathogens 

Ning et al. [46] developed a label-free amperometric immunosensor for rapid determination of HIV p24 protein using magnetic bioconjugates of a specific antibody and Fe_3_O_4_ (core)/Au (shell) nanoparticles-coated multiwalled carbon nanotubes, which were magnetically captured on the surface of *N,N″*-bis-(2-hydroxy-methylene)-*o*-phenylenediamine copper (CuRb)-modified SPCEs. The DPV cathodic current arising from the reduction of H_2_O_2_ catalyzed by CuRb decreased linearly with the HIV p24 protein concentration in the 0.6–160 μg L^−^^1^ range. The immunosensor provided a LOD (3σ) of 0.32 μg L^−^^1^ and results in agreement with the ELISA methodology in the analysis of HIV patients’ serum samples. 

Gan et al. developed another amperometric immunosensor for the determination of HIV p24 by immobilizing the capture antibody onto a gold electrode modified with poly(L-lysine) and mercaptosuccinic acid stabilized Fe_3_O_4_(core)/gold(shell) nanoparticles multilayer films [47]. The DPV response corresponding to the electron transfer inhibition of [Fe(CN)_6_]^3−/4−^ in the presence of antigen allowed a wide detection range (0.1–100.0 ng mL^−1^), a LOD (S/N = 3) of 0.05 ng mL^−1^ and successful applicability to the determination in human serum specimens. 

Another sandwich immunosensor for HIV p24 protein involved an HRP-labeled detector antibody [48] and electroplating of AuNPs onto a GCE surface by chronoamperometry to enhance the conductivity of the electrode and facilitate the immobilization of a large amount of capture antibodies while keeping their biological activity. Using DPV in the presence of H_2_O_2_ and hydroquinone (HQ), the immunosensor showed a linear relationship with the concentration of HIV p24 protein between 0.01 and 100 ng mL^−1^, and a LOD (3σ) of 0.008 ng mL^−1^. Interestingly, the method provided a sensitivity two orders of magnitude larger for standard solutions and comparable results in the analysis of serum samples compared to the standard ELISA method. 

Gan et al. [49] proposed another electrochemical sandwich immunosensor for p24 antigen by immobilizing the capture antibody on silicon dioxide-coated magnetic Fe_3_O_4_ nanoparticles, and using a detector antibody immobilized on gold nanocolloids and a novel copolymer of an EnVision regent (EV, a dextrin amine skeleton anchoring more than 100 molecules of HRP and 15 molecules of antiIgG) as signal tag. The DPV signal obtained with the system HQ/H_2_O_2_ was monitored after magnetically capturing the MNPs–immunocomplexes on the SPCE surface. The immunosensor offered a linear concentration range of 0.001–10.00 ng mL^−1^, which is a 1000 times higher sensitivity than the ELISA method, a LOD of 0.5 pg mL^−1^ and successful applicability for the analysis of spiked serum samples. 

A MBs-based electrochemical immunosensor for pseudorabies virus (PRV) antibody detection was proposed by Li et al. [50]. In this approach, MBs were modified with PRV antigen and used for selective capture of the specific antibodies in swine serum, which were labeled in a further step with a secondary antibody modified with AuNPs. After magnetic capture of the immunocomplex-coated MBs and electro-oxidation of the AuNPs to produce AuCl_4_^−^, the electrochemical detection was carried out by DPV measuring the reduction current of gold. The DPV signal was linear with the standard PRV antibody positive serum dilution over the 1:1000–1:250 range, and the LOD (S/N = 3) was estimated as 1:1000. The applicability of the immunosensor was evaluated through the analysis of 52 swine serum samples, providing results in agreement with those obtained using a standard ELISA kit.

Bhimji et al. [51] developed a simple electrochemical immunoassay for the detection of HIV-1 and HIV-2 antibodies by attaching covalently specific HIV-1 and HIV-2 antigenic peptides to a SU-8 substrate, a negative epoxy-based photoresist originally developed at IBM Research and ideal to be functionalized with biomolecules without any pretreatment due to the presence of exposed epoxy groups. The captured HIV antibodies were labeled with an AP-conjugated secondary antibody and the oxidation of p-aminophenol generated by hydrolysis of p-aminophenyl phosphate by AP was measured by DPV. Linearity in the response was found over a wide concentration range (0.001–1 μg mL^−^^1^) with LODs of 1 ng mL^−^^1^ (6.7 pM) for both HIV-1 and HIV-2 antibodies. The LODs were calculated as follows: LoD = LoB + 1.645(SD_low concentration sample_), where LoB = mean blank + 1.645(SD_blank_). The clinical applicability of the method was demonstrated by analyzing HIV patient clinical samples. 

An integrated immunosensor for detection of antibodies against hemagglutinin (HA) from avian influenza virus H5N1 was developed by Jarocka et al. [52]. The method involved immunorecognition of the recombinant His-tagged HA (His6-H5 HA) by anti-His IgG monoclonal antibody previously immobilized through its Fc region onto ProtA-modified GCE. By measuring the decrease of the Cu(II) redox processes after target antibodies binding using Osteryoung square-wave voltammetry (OSWV), this immunosensor displayed a linear range of 4–20 pg mL^−1^, a LOD (3.3 × s_b_/m) of 2.1 pg mL^−1^ and a 10^4^ times better sensitivity than the ELISA methodology. Determinations in hen sera samples provided results in agreement with the conventional ELISA methodology. Interestingly, the immunosensor was able to discriminate between sera of non-vaccinated and vaccinated chickens against the target virus. The same authors also developed another strategy for the detection of the same anti-HA antibodies by oriented immobilization of the recombinant His-tagged HA onto a mixed layer containing the thiol derivative of dipyrromethene (DPM) with Cu(II) ions complexed (DPM–Cu(II) complex) and 4-mercapto-1-butanol [53]. The antigen–antibody binding was followed by square-wave voltammetry, allowing a LOD (3.3 × s_b_/m) of 2.4 pg mL^−1^ and a dynamic range of 4.0–100.0 pg mL^−1^. In addition, the method was applied to the detection of antibodies in hen sera from individuals vaccinated and non-vaccinated against the avian influenza virus type H5N1, providing a sensitivity 20–200 times better than the ELISA methodology.

**Figure 10:**
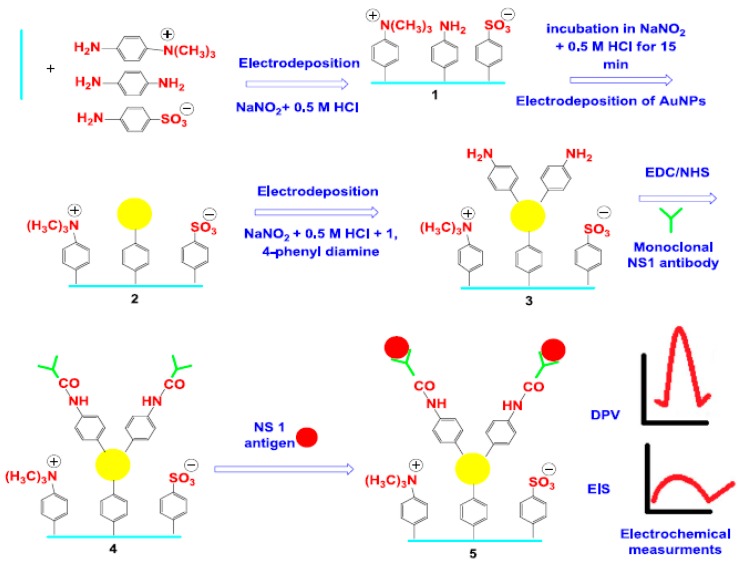
Schematic display of the electrochemical immunosensor involving the use of zwitterionic antifouling molecules, AuNPs and capture antibodies for direct detection of the Dengue Virus NS1 biomarker: (1) electrodeposition of aryldiazonium cations on the ITO electrode; (2) electrodeposition of AuNPs; (3) electrodeposition of aryldiazonium cations onto AuNPs; (4) covalent immobilization of NS1 antibody and (5) antigen recognition and electrochemical detection. Reprinted from [54] with permission.

Darwish et al. [54] developed a sensitive label-free electrochemical immunosensor for the unstructured protein NS1 (a Dengue virus biomarker) determination. The immunosensing scaffold consisted of an ITO electrode modified with aryl-diazonium-cation-derived zwitterionic antifouling molecules (4-sulfophenyl, 4-trimethylammoniophenyl and 1,4-phenylenediamine) capture antibodies and AuNPs (Figure 10). This immunosensor, is based on measuring the increase in the electron transfer resistance after antigen binding by electrochemical impedance spectroscopy (EIS) in the presence of Fe(CN)_6_^3^^−^/Fe(CN)_6_^4^^−^. It exhibited a wide detection range of 5–4000 ng mL^−1^ and feasibility to detect the target antigen in serum specimens from Dengue virus infected patients. 

**Figure 11:**
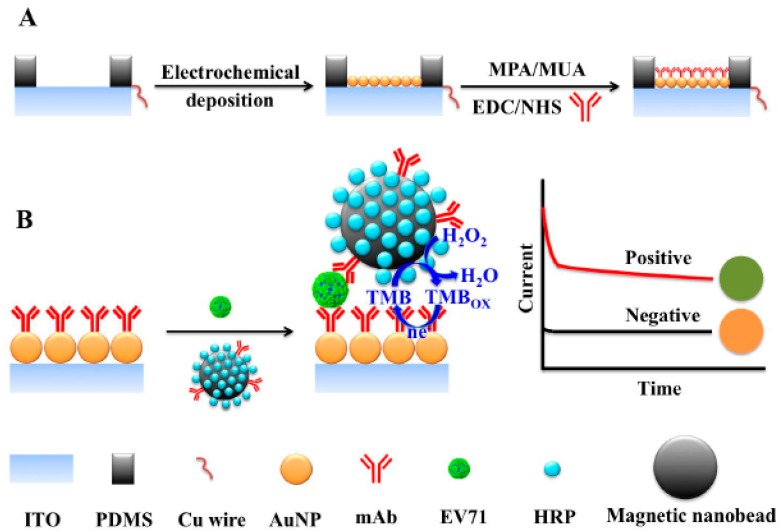
(**A**) Schematic illustration of the preparation of monoclonal antibody-modified AuNPs/ITO electrodes by electrochemical deposition of AuNPs followed by assembling of a mixed SAM and covalent immobilization of the capture antibody; (**B**) electrochemical immunosensing for EV71 detection by recognition of the antigen and its further labeling with magnetic nanobeads dually-labeled with HRP and TMB as the redox mediator. Reprinted from [55] with permission.

Very recently, an electrochemical immunosensor for the detection of human enterovirus 71 (EV71), the major pathogen causing hand, foot and mouth disease (HFMD) among children, has been reported [55]. The EV71 virions from the sample were selectively captured on a specific monoclonal antibody-modified AuNPs-coated ITO electrode, and labeled with magnetic nanobeads modified with the detector antibody and HRP (Figure 11). By chronoamperometric monitoring of the reduction of oxidized TMB in the presence of H_2_O_2_, a LOD (S = B + 3σ) of 0.01 ng mL^−1^ was achieved. The immunosensor demonstrated successful applicability to the analysis of serum samples providing results in agreement with those obtained by reverse transcription-polymerase chain reaction (RT-PCR).

**Figure 12:**
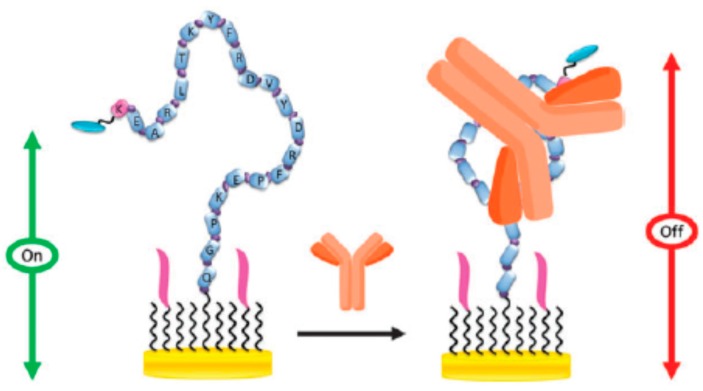
Electrochemical determination of HIV antibodies at a gold electrode modified with a thiolated specific peptide and short DNAs as diluents. Reprinted from [56] with permission.

McQuistan et al. [56] developed an electrochemical peptide-based sensor for HIV antibodies by self-assembling a specific thiolated peptide labeled with methylene blue (MB) and short thiolated DNAs on gold electrodes (Figure 12). The recognition of the HIV antibody limited the probe mobility, and the MB reduction peak measured by alternating current voltammetry decreased. This platform demonstrated excellent antifouling properties due to the short DNA diluents, a LOD of 1 nM and promising applicability for the determination of the antibodies in spiked 10% saliva samples. 

A sensitive and selective label-free electrochemical DNA biosensor for the detection of specific dengue virus serotype 3 (DENV-3) sequences was developed by adsorbing a 22-nts DNA probe on a pencil graphite electrode (PGE) [57]. Measurement of the electrochemical oxidation of guanine by DPV allowed a linear range between 10 and 100 nM, a LOD (3 × s_b_/m) of 3.09 nM and successful performance in serum samples.

Bartosik et al. [58] proposed a MBs-based approach for the detection of DNA sequences from high-risk human papillomavirus (hrHPV) strains (HPV-16 and HPV-18) using a sandwich hybridization format, where target HPV DNA is captured using the specific DNA capture probe-modified MBs and sandwiched with a digoxigenin-labeled detector probe. The resulting sandwich duplexes were further labeled with an antidigoxigenin-HRP antibody, and upon magnetic capture of the resulting modified MBs, chronoamperometric detection was performed at a screen-printed 8-carbon electrodes array using the H_2_O_2_/HQ system. The method exhibited a linear range of 1 pM to 1 nM, a LOD (S = B + 3σ) of 1 pM, selective discrimination between HPV-16 and HPV-18 strains and feasible application to the analysis of both cancer cell lines and real patient cervical brush smears, providing results which correlated well with standard methods.

Very recently, a highly sensitive impedimetric biosensor for detecting human norovirus has been proposed by assembling a thiolated affinity peptide on a gold electrode [59]. The impedimetric peptide sensor showed a wide linear concentration range (10^1^–10^7^ copies mL^−1^) and LODs of 99.8 nM for recombinant noroviral capsid proteins and 7.8 copies mL^−1^ for real norovirus from diarrheal patients. The feasibility to perform the determination in fetal bovine serum was also demonstrated. 

## 5. General Considerations, Challenges and Future Prospects

Inappropriate antimicrobial use, proliferation of multidrug-resistant pathogens, emergence of new infectious agents and ease of rapid disease dissemination due to overpopulation and globalization are among the major challenges for management of infectious diseases. Within this context, timely diagnosis and initiation of targeted antimicrobial treatment are essential for clinical management of infectious diseases in a successful way [2].

Current diagnosis of clinically relevant infectious diseases caused by bacterial and viral pathogens relies on a variety of laboratory-based tests which include microscopy, culture, immunoassays and nucleic-acid amplification. These in vitro diagnostics methods, while widely used, have well-recognized shortcomings. While culture has a significant time delay, microscopy lacks sensitivity in many clinical scenarios. Immunoassays, such as ELISA, are highly sensitive, but labor intensive and challenging to implement for multiplexing detection. Nucleic-acid amplification tests, such as PCR, although offering molecular specificity, require complex sample preparation, are susceptible to false positive results and are difficult to integrate in easy-to-use devices for on-site detection [2].

Nowadays, healthcare systems are pursuing the development of cheap, rapid, non-invasive, portable and user-friendly tools able to provide multiple results from a single sample, even in remote settings, in order to improve patient diagnosis, treatment, monitoring and management [1].

In this sense, electrochemical biosensors provide an easy-to-use, sensitive and inexpensive technology, with the ability to identify pathogens rapidly and predict effective treatment using minimally invasive approaches. Other advantages include small volume manipulation (less reagents and lower cost), easy and rapid experimental protocols, low energy consumption, portability, high-throughput, multiplexing ability, reasonable cost and easy tailoring for the needs of low- and middle-income countries. Most of these electrochemical biosensors are superior in comparison with the traditional methods. They have a comparable or better sensitivity and selectivity, with no need for longer and complex protocols or very expensive equipment. Rapid development of nanotechnology, which has opened a new way for construction of biosensors with even better features, is moving this field quickly toward its destination.

The methods highlighted in this review involve the use of a wide variety of bioreceptors, assay formats and electrochemical techniques used in the development of electrochemical affinity sensors for detection of infectious bacteria and virus. Thee reported methods have been implemented using integrated or MBs-based formats, conventional or screen-printed electrodes and with and without labels.

Regarding infectious bacteria, although a few sandwich immunosensors have been developed for the determination of whole bacteria or specific surface bacterial proteins, most of the highlighted methods make use of nucleic acid-based biosensors targeting bacterial 16S rRNA and specific regions of characteristic genes. In these methods, the achievement of the required sensitivity led to the use of gold scaffolds modified with multicomponent SAMs involving thiolated capture probes or nanostructured with DNATH or the coupling with conventional (PCR) and isothermal (HDA and RPA) nucleic acid amplification strategies. Conversely, most of the approaches reported for the detection of infectious viruses (considerably less numerous than those described for bacteria) utilize electrochemical immunosensors to detect viral-specific proteins or autoantibodies against them. Less frequently, immunosensors for whole virions and DNA sensors for the detection of specific sequences have also been proposed. 

Despite the impressive research output in recent years and the promising capabilities demonstrated by electrochemical affinity biosensors for the determination of circulating biomarkers related tp bacterial and viral pathogens, the manufacture of commercially available systems for real world settings is seriously lagging behind due to important issues that should be overcome. The high levels of sensitivity and specificity attained in the selected papers must be proven with highly heterogeneous samples representative of clinical specimens. This is especially relevant in infectious disease diagnosis where analyte concentrations can vary from femto- to picomolar levels, highlighting the importance of the dynamic range. The biomolecular targets can be outnumbered by a million-fold excess of non-target species, making clinical specimen analysis challenging. Moreover, in order to bring laboratory-based biosensor systems to market, a closer and more active collaboration among academies, healthcare units and industries is required for the successful realization of real LOCT devices and to address other key issues. These include careful assessment of suitability of the base material (inexpensive, reproducible, electrochemically favorable and chemically stable) for particular application needs, sample preparation, matrix effects, reliable clinical validation using a large number of real patient samples, appropriate functionality independent of production and environmental variables, long-term storage stability, multiplexing capabilities and full integration in automated and miniaturized systems. Although the potential of electrochemical affinity biosensors to benefit POC diagnostics has been demonstrated for decades, it is unlikely that integrated lab-on-a-chip systems, able to directly deal with raw samples, will be on the market in the next five years. Indeed, the driving force for commercialization fully relies on the cost–effectiveness delivered by the microfluidics technology, which involves, apart from the cost the real clinical benefits delivered such as the development of universal integrated systems, the ability to efficiently handle a wide variety of clinical samples such as urine, blood and saliva for different infectious viruses or bacteria [2]. 

Additionally, the development and exploration of novel bioreceptors, such as bacteriophages, artificial proteins and engineered antibodies, constitutes alternatives with improved chemical and biological stability and binding efficiencies and the ability to be produced with ease and precise control. Therefore, these novel bioreceptors will play a determinant role for improving the performance of the resulting electrochemical biosensors paving the way for their commercialization. 

These important and challenging issues mean that it is most likely that several years will pass before electrochemical affinity biosensing devices may fully replace techniques currently used for the detection and quantification of infectious pathogens in biofluids. Nevertheless, the market demand and research trends presented in this review clearly demonstrate the eliciting considerable excitement of the electrochemical biosensing diagnostic platforms as they promise a paradigmatic shift in the way to conduct disease diagnosis and health monitoring in the near future. The development and deployment of these smart biosensing systems will have a profound impact leading to more rapid clinical decision making by providing appropriate and timely antimicrobial and antiviral treatment with the corresponding patient stress reduction, antibiotic-resistant infections and healthcare costs. Moreover, constant developments in nanoinstrumentation and molecular biology, nanofabrication and labeling methods will lead to more sensitive, accurate and rapid, handheld and user-friendly electrochemical biosensors with multiplexing capabilities, which will find an important parcel for non-invasive, more effective and timely POC diagnostics of infectious pathogens.
